# Mental health among healthcare workers during the prolonged COVID-19 pandemic: A cross-sectional survey in Jilin Province in China

**DOI:** 10.3389/fpubh.2022.1030808

**Published:** 2022-10-17

**Authors:** Liangwen Ning, Huanhuan Jia, Jianxing Yu, Shang Gao, Panpan Shang, Peng Cao, Xihe Yu

**Affiliations:** ^1^School of Public Administration, Jilin University, Changchun, China; ^2^School of Public Health, Jilin University, Changchun, China

**Keywords:** COVID-19, healthcare worker, mental health, DASS-21, cross-sectional survey

## Abstract

**Background:**

The prolonged COVID-19 pandemic has seriously impacted the mental health of healthcare workers. This study aimed to explore the mental health status of healthcare workers, compare the differences in mental health between physicians and nurses, and verify the impact of risk perception on mental health in the long-term COVID-19 pandemic in Jilin Province, China.

**Methods:**

A stratified random sample was used to conduct an on-site questionnaire survey in December 2020 to measure the mental health status, risk perceptions, and demographic characteristics of healthcare workers in Jilin Province, China. A total of 3,383 participants completed the questionnaire survey, of which 3,373 were valid questionnaires.

**Results:**

A total of 23.6% (*n* = 795) of participants had symptoms of depression, 27.4% (*n* = 923) had symptoms of anxiety, and 16.3% (*n* = 551) had symptoms of stress. Physicians reported significantly higher rates of depression and anxiety than nurses (*p* = 0.023, *p* = 0.013, respectively). There was no significant difference in the proportion of participants with stress between physicians and nurses (*p* = 0.474). Multivariate logistic regression results showed that healthcare workers who had a high level of risk perception were more likely to have symptoms of depression (AOR = 4.12, *p* < 0.001), anxiety (AOR = 3.68, *p* < 0.001), and stress (AOR = 4.45, *p* < 0.001) after controlling for other variables.

**Conclusion:**

At least one in six healthcare workers experienced mental health problems, and physicians were more likely than nurses to suffer from depression during the prolonged COVID-19 epidemic. Risk perception was highly predictive of depression, anxiety, and stress symptoms in medical staff. Public health interventions are needed to mitigate the long-term psychological impact of the COVID-19 pandemic.

## Introduction

The outbreak of the COVID-19 pandemic in early 2020 continues to threaten societies all over the world and has had a major impact on health systems (1). The World Health Organization (WHO) declared the COVID-19 pandemic a Public Health Emergency of international concern in January 2020 (2), and researchers generally agreed that the outbreak of COVID-19 is likely to be the worst pandemic since the 1918 influenza pandemic (3). COVID-19 continues to spread internationally, with the totals for infections and deaths rising. How governments and communities around the world have responded to the COVID-19 pandemic has varied widely (4). Studies have shown that the pandemic is still an ongoing major public health challenge (5).

The mental health status of health professionals has attracted much attention during the COVID-19 pandemic. Previous studies have demonstrated that COVID-19 has introduced a global macrostressor that has a major negative influence on the mental health of populations worldwide (6), and many studies have shown that the impact of COVID-19 on the mental health of medical staff has been more severe than that of the general public (7). Scientific evidence has revealed that healthcare workers, especially those on the front-line of the epidemic, have endured enormous psychological pressure during the COVID-19 pandemic because of increased workload, the risk of exposure to COVID-19, fatigue, burnout, stigma, etc. (8). In addition, the risk effect has been amplified due to extensive media coverage that may increase the perception of risk among medical staff. The perceived and actual need for healthcare workers to go to the front lines of the epidemic to support prevention efforts, resulting in a break in the routine work style, may further increase their mental health burdens (9). A study conducted in the UK and the US found that front-line healthcare workers had increased risk of contracting COVID-19 compared to the general populations (10). Previous studies have observed fatigue, decreased cognitive function and job performance, stress, crying, suicidal intention and other problems (11, 12). Lai et al.'s survey of healthcare workers during the outbreak in Wuhan showed that the proportions of respondents reporting symptoms of depression, anxiety, insomnia, and suffering were 50.4, 44.6, 34.0, and 71.5%, respectively (13). Female nurses on the front-lines working in Wuhan, China, reported more severe measures of all mental health symptoms than other healthcare workers. The negative impact on healthcare workers does not only affect the prevention and control of COVID-19 but may also lead to other serious consequences, such as lower morale of healthcare workers, lower job satisfaction, higher absenteeism, and lower quality of medical services or treatment (14, 15). The psychological problems of healthcare workers in the context of a pandemic have become a focus of attention for scholars and health departments, and the protection against psychological problems in healthcare workers during a pandemic has become an important issue.

Few studies have looked at the long-term effects of infectious diseases on the mental health of health care workers, but the results have been inconsistent. Wu et al. found that usual ward nurses were more prone to burnout during the epidemic than frontline nurses, suggesting the need to pay attention to medical staff who deal with COVID-19 daily during this crisis (16). Similarly, Lee et al. found a significant increase in mental health problems among healthcare workers a year after the SARS outbreak (17). The longitudinal study by Cai et al. of Chinese medical personnel showed that depression, anxiety, and posttraumatic stress disorder symptoms were significantly higher during the outbreak than during the stabilization of the outbreak (18). However, Zhou's longitudinal study of healthcare workers on emergency medical assistance teams supporting Hubei Province found that healthcare workers were in a worse mental state after returning to their hometown (19). The results from another year-long longitudinal study of health workers in emergency departments in Singapore by Th'ng et al. showed significant improvement in anxiety symptoms and a significant increase in depressive symptoms 1 year after the outbreak (20).

Several studies have focused on differences in psychological problems between physicians and nurses during the COVID-19 pandemic, and most of these findings suggest that nurses are prone to more severe mental health problems during the current outbreak (21–23). A study of Belgian health professionals found that 63.2% of nurses reported symptoms of anxiety compared to 23.5% of doctors (24). However, a few studies reflect inconsistent findings. A cross-sectional survey conducted by Wang and colleagues in four hospitals in Guangdong Province, China, showed that physicians were more likely to suffer from moderate or severe depression than nurses (25). A longitudinal study in Singapore showed an increased prevalence of depression among a population of physicians in emergency departments in 2021 compared with a year earlier, and also showed higher total depression scores in this population than nursing staff (20). A comprehensive understanding of the vulnerability of healthcare workers' mental health in the context of the COVID-19 pandemic is critical for the development of relevant preventative and social policies during a pandemic. It is necessary to continue to compare the differences in mental health issues between physician and nurse populations during the prolonged COVID-19 pandemic.

According to cognitive assessment theory, risk perception can be considered a form of threat assessment and thus a determinant of mental health responses (26). According to the psychometric paradigm of Slovic, risk perception has two dimensions, “fear” and “unknown”(27), which are exacerbated in healthcare workers by the prevalence of COVID-19. A large body of previous research from psychology, clinical medicine, and economics suggests that risk perceptions often drive emotional and psychological distress (28). Several studies have assessed risk perceptions associated with COVID-19 and mental health. Ding et al. found that risk perceptions of COVID-19 were associated with levels of depression (29). Teufel et al. observed similar levels of risk perception and levels of COVID-19-related fear, depression, and generalized anxiety (30). However, while some studies have suggested an association between risk perception and mental health, others have questioned whether this association can be attributed to differences in sample selection, methodology, and social context between studies (31). In addition, previous studies mainly focused on the general public, and paid little attention to the association between risk perception and mental health among medical staff. Therefore, there is a need to further explore the relationship between risk perceptions and mental health among medical staff during the prolonged COVID-19 epidemic.

The Joint WHO-China 2019 report on the Coronavirus Disease Mission from February 16 to 24, 2020, suggests that China has begun to return to normal (32). According to statistics from the National Health Commission of China (33), Jilin Province had new cases in February, May, and July following the first confirmed cases announced on January 22, 2020. The number of confirmed cases in July reached 138, the highest in the whole year. Subsequently, the epidemic crisis in Jilin Province ended and there were no further outbreaks by the end of the year. Despite the absence of new cases in Jilin Province during this period, there are still clusters or scattered outbreaks of cases in other Chinese provinces and cities as well as globally. Jilin Province continues to face potential threats and pressures, and healthcare workers remain in a highly stressful state of risk preparedness. Therefore, we conducted a study during the regular prevention and control of the COVID-19 epidemic in Jilin Province to achieve three research objectives: (1) investigating the prevalence of the mental health among healthcare workers, (2) comparing the differences in mental health between physicians and nurses in China, and (3) exploring the impact of risk perception on mental health.

## Materials and methods

### Design and sample

Most data collection efforts on healthcare workers' mental health used online surveys to obtain samples, because of the COVID-19 pandemic. Despite the strengths of flexibility, speed, timeliness, convenience, etc., online surveys still have unavoidable weaknesses, such as sample selection bias, and low implementation and response rates, which may have some effect on sample representativeness (34). This study conducted an on-site cross-sectional survey of medical staff in public hospitals in Jilin Province from December 1 to December 30, 2020. First, a stratified sampling method was used to divide all public hospitals in Jilin Province into municipal public hospitals and county public hospitals. Since urban public hospitals are more clustered, 25% of public hospitals were randomly selected according to their region, type, and level. Since counties are more dispersed and public hospitals at the county level are more heterogeneous, one public general hospital and one public TCM hospital were randomly selected in each county. Ultimately, 29 municipal public hospitals and 80 county-level public hospitals were included in the study sample. Then, 20 doctors and 10 nurses were selected from each hospital for the on-site survey using a quota sampling method. The criteria for inclusion in this study were: in-service physicians and nurses between 18 and 60, were able to complete the questionnaire on their own and agreed to participate in the study. The exclusion criteria for participants were: physicians and nurses who were on leave during the period of investigation; did not want to participate in the study and were supporting other regions due to the COVID-19 pandemic.

### Sample size

We used PASS 15 to estimate the study sample. we calculated a sample size value of 2449, assuming that the 50% of healthcare workers have mental health problems and setting the confidence level at 95% and the margin of error at 2%. Considering the non-response rate and missing values, the final sample size was inflated by 20% to be 3061.

### Data collection

Our study was approved by the Medical Ethics Committee of Jilin University and IRB code is No. 2019-12-03. The purpose and protocol of the study were clearly explained by the investigator at the beginning of the survey. Participants had to agree to the study statement before starting the questionnaire. In total, 3,383 people participated the questionnaire. The collected questionnaires were verified and 10 questionnaires with logical errors were excluded, resulting in a valid sample size of 3373 (99.7% of the returned questionnaires) for inclusion in the study.

### Measurement

#### Demographic variables

The demographic variables in this study included hospital location, gender, age, marital status, education level, department, professional title, working years, average monthly income, and whether or not they were exposed to COVID-19 positive patients. Previous studies suggest an association between demographic variables and mental health in the COVID-19 epidemic.

#### Risk perception

Risk perception was measured with a scale based on a previous study during the SARS outbreak in 2003 to measure healthcare workers' threat perception of COVID-19-related risks (35). The scale consists of 10 items such as “I believed that my job poses a great risk to me” which were rated on a 5-point Likert scale (1 = completely disagree and 5 = completely agree). The language of Risk Perception Scale is Chinese and the results of reliability and validity analysis show that the risk perception scale had good reliability (Cronbach's = 0.870) and validity (RMSEA = 0.985, GFI = 0.986, TLI = 0.957). The average score of all items above 3 was deemed high in risk perception.

#### Mental health

We measured depression, anxiety and stress to assess the mental health of medical staff during the COVID-19 epidemic. Depression is a condition characterized by a sad mood, low self-esteem, apathy, and when severe, suicidal impulses; while anxiety often manifests itself as excessive worry, hypervigilance; symptoms of stress are usually associated with excitement or tension as a result of a lack of coping strategies (36).

The Chinese version of the Depression Anxiety and Stress Scale (DASS-21 scale) was used in this study to assess the prevalence of depression, anxiety, and stress among healthcare workers in China. The DASS-21 scale was originally developed by Lovibond (36), and Gong developed a Chinese version of the scale based on it (37). The scale has been used in several studies in China during the COVID-19 epidemic (38, 39). In this study, the Cronbach's α of the total DASS-21 scale was 0.971, indicating that the scale has good reliability. The results of the confirmatory factor analysis indicated that the scales had good validity (CFI = 0.984, TLI = 0.976, RMSEA = 0.049).

The scale contains 3 subscales, and each subscale comprises seven items covering depression, anxiety, and stress. Items on the depression scale assess symptoms of dysphoric mood, and example items include “I could not seem to experience any positive feeling at all.” Items on the anxiety scale measure symptoms pertaining to physiological hyperarousal, such as “I was aware of dryness of my mouth.” Items on the stress scale evaluate negative affectivity, such as, “I found it hard to wind down.” A 4-point Likert scale was used for all responses (0 = never a problem, 1 = sometimes a problem, 2 = often a problem, and 3 = almost always a problem).

We multiplied each score by two for comparison with the original 42 items of the DASS scale (40). The total score of each dimension was categorized as “normal,” “mild,” “moderate,” “severe,” and “extremely severe,” according to the DASS manual. On the depression scale, 0–9 indicates normal depression, 10–13 indicates mild depression, 14–20 indicates moderate depression, 21–27 indicates severe depression and 28–42 indicates extremely severe depression. On the anxiety scale, 0–7 indicates normal, 8–9 indicates considered mild anxiety, 10–14 indicates moderate anxiety, 15–19 indicates severe anxiety and 20–42 indicates extremely severe anxiety. On the stress scale, 0–14 indicates normal, 15–18 indicates mild stress, 19–25 indicates moderate stress, 26–33 indicates severe stress and 34–42 indicates extremely severe stress. Participants who fell into the “mild” or higher category were identified as experiencing symptoms of depression, anxiety, or stress.

### Statistical analysis

Our study described the characteristics of the study participants by frequency analysis. The mean and standard deviation (SD) of the scores for each risk perception entry were calculated, and the physician and nurse groups were compared using independent *t*-test. A chi-square test was used to test for differences in the prevalence of depression, anxiety, and stress symptoms between the physician and nurse groups.

Participants were divided into two groups: those who suffered from symptoms of depression, anxiety, or stress, and those who did not. A chi-square test was used to compare significant differences between different demographic characteristics and depression, anxiety, and stress. Three logistic regression models were developed to identify predictors of depression, anxiety, and stress symptoms. Variables related to sociodemographic characteristics, work-related variables, and risk perception variables were entered into the regression models. To test the robustness of the results of the logistic regression model, we developed a linear regression model that treated depression, anxiety, and stress symptom scores as a continuous variable (Supplementary material).

IBM SPSS Statistics 25 programs were used for statistical analysis.

## Results

### Demographic characteristics of respondents

As shown in Table 1, 63.7% of the sample were doctors, and 36.3% were nurses in total. Most of the respondents were female (69.8%), 31–45 years old (52.9%), married (79.8%), had a bachelor's degree (61.2%), had a junior or not-professional rank (44.4%), had <10 years of work experience (45.3%) and had a monthly income of 5,000 yuan or less (63.3%).

**Table 1:** Sociodemographic characteristics of study participants.

**Characteristics**	**Total**		**Doctors**		**Nurses**	
	***n* = 3373**	**%**	***n* = 2149**	**%**	***n* = 1224**	**%**
**Hospital location**						
Urban	867	25.7	569	26.5	298	24.3
County	2506	74.3	1580	73.5	926	75.7
**Gender**						
Male	1018	30.2	991	46.1	27	2.2
Female	2355	69.8	1158	53.9	1197	97.8
**Age**						
18–30	832	24.7	424	19.7	408	33.3
31–45	1784	52.9	1171	54.5	613	50.1
>45	757	22.4	554	25.8	203	16.6
**Marital status**						
Unmarried/Divorced/widowed	680	20.2	418	19.5	262	21.4
Married	2693	79.8	1731	80.5	962	78.6
**Education level**						
Junior college or below	1010	29.9	523	24.3	487	39.8
Bachelor's degree	2065	61.2	1361	63.3	704	57.5
Master degree or above	298	8.8	265	12.3	33	2.7
**Health care unit**						
Internal Medicine	1175	34.8	803	37.4	372	30.4
Surgery	641	19.0	394	18.3	247	20.2
Obstetrics and Gynecology	210	6.2	126	5.9	84	6.9
Pediatrics	154	4.6	99	4.6	55	4.5
Chinese medicine	142	4.2	125	5.8	17	1.4
Public health section	18	0.5	11	0.5	7	0.6
Other sections (Laboratory, etc.)	1033	30.6	591	27.5	442	36.1
**Professional rank**						
Junior/No	1498	44.4	822	38.3	676	55.2
Middle	1034	30.7	700	32.6	334	27.3
Senior	841	24.9	627	29.2	214	17.5
**Working years**						
< 10	1528	45.3	998	46.4	530	43.3
10–20	1131	33.5	680	31.6	451	36.8
>20	714	21.2	471	21.9	243	19.9
**Average monthly income (CNY)**						
≤ 5000	2136	63.3	1239	57.7	897	73.3
>5000	1237	36.7	910	42.3	327	26.7
**Exposure to confirmed or suspected cases**						
Yes	284	8.4	175	8.1	109	8.9
No	3089	91.6	1974	91.9	1115	91.1

### Prevalence of depression, anxiety, and stress symptoms in the sample

Table 2 demonstrates the percentages of healthcare workers who experienced various levels of symptoms of depression, anxiety, and stress. In total, 23.6% (*n* = 795) of the respondents had symptoms of depression, 27.4% (*n* = 923) of participants had symptoms of anxiety, 16.3% (*n* = 551) of participants had symptom of stress.

**Table 2:** Prevalence of depression, anxiety and stress symptoms in the sample.

	**Total**		**Doctor**		**Nurse**		***p***
	***n***	**%**	***n***	**%**	***n***	**%**	
**Depression**							0.023
Normal	2578	76.4	1607	74.8	971	79.3	
Mild	191	5.7	133	6.2	58	4.7	
Moderate	361	10.7	250	11.6	111	9.1	
Severe	98	2.9	60	2.8	38	3.1	
Extremely severe	145	4.3	99	4.6	46	3.8	
**Anxiety**							0.013
Normal	2450	72.6	1537	71.5	913	74.6	
Mild	120	3.6	70	3.3	50	4.1	
Moderate	337	10.0	242	11.3	95	7.8	
Severe	139	4.1	93	4.3	46	3.8	
Extremely severe	327	9.7	207	9.6	120	9.8	
**Stress**							0.474
Normal	2822	83.7	1785	83.1	1037	84.7	
Mild	152	4.5	105	4.9	47	3.8	
Moderate	157	4.7	97	4.5	60	4.9	
Severe	141	4.2	95	4.4	46	3.8	
Extremely severe	101	3.0	67	3.1	34	2.8	

In addition, Table 2 also shows statistically significant differences in the proportions of different levels of depression and anxiety symptoms between the doctors and nurse groups, with significantly more physicians reporting depression and anxiety than nurses (*p* = 0.023, *p* = 0.013, respectively). There was no significant difference in the proportion of participants with stress between doctors and nurses (*p* = 0.474).

### Risk perception of respondents

Table 3 shows the risk perception scores of the healthcare workers. On the risk perception sections of the survey, a total of 596 (17.7%) respondents gave a rating higher than 3 out of a possible score of 5. A total of 379 (17.6%) doctors had a high level of risk perception about COVID-19, and 217 (17.7%) nurses had a high level of risk perception. No significant differences were found for perceived risk between doctors and nurses (*p* = 0.946).

**Table 3:** Risk perception of respondents.

	**Risk perception scores (M**±**SD)**				**Risk perception score** >**3 [*****n*** **(%)]**			
	**Total**	**Doctor**	**Nurse**	***p***	**Total**	**Doctor**	**Nurse**	***p***
1. I believed that my job poses a great risk to me	2.86 ± 1.23	2.87 ± 1.23	2.86 ± 1.23	0.810	1019 (30.2)	643 (29.9)	376 (30.7)	0.627
2. I felt extra stress at work	2.82 ± 1.23	2.86 ± 1.23	2.73 ± 1.23	0.003	1015 (30.1)	681 (31.7)	334 (27.3)	0.007
3. I was afraid of falling ill with COVID-19	2.87 ± 1.41	2.84 ± 1.39	2.91 ± 1.42	0.188	1155 (34.2)	712 (33.1)	443 (36.2)	0.072
4. I often worried about whether I am infected	2.25 ± 1.23	2.23 ± 1.22	2.28 ± 1.26	0.336	577 (17.1)	350 (16.3)	227 (18.5)	0.094
5. I thought I may not survive if I got COVID-19	1.70 ± 1.03	1.70 ± 1.03	1.71 ± 1.03	0.742	253 (7.5)	160 (7.4)	93 (7.6)	0.871
6. I have thought about resigning because of COVID-19	1.31 ± 0.73	1.33 ± 0.78	1.26 ± 0.65	0.007	100 (3.0)	74 (3.4)	26 (2.1)	0.030
7. I was afraid I would pass COVID-19 on to others	2.40 ± 1.37	2.36 ± 1.37	2.45 ± 1.38	0.065	771 (22.9)	475 (22.1)	296 (24.2)	0.167
8. My family and friends are worried that I will infect them	2.14 ± 1.26	2.16 ± 1.26	2.10 ± 1.25	0.188	550 (16.3)	349 (16.2)	201 (16.4)	0.891
9. People avoided my family because of my work	1.71 ± 1.04	1.74 ± 1.06	1.66 ± 1.00	0.027	268 (7.9)	184 (8.6)	84 (6.9)	0.079
10. I was at risk of contacting COVID-19 patients in the hospital	2.97 ± 1.38	2.96 ± 1.36	2.99 ± 1.41	0.533	1265 (37.5)	782 (36.4)	483 (39.5)	0.076
Total scores	23.02 ± 8.18	23.06 ± 8.16	22.96 ± 8.22	0.716	596 (17.7)	379 (17.6)	217 (17.7)	0.946

### Univariate analysis of symptoms of depression, anxiety, and stress

As shown in Table 4, univariate analysis demonstrated that hospital location, education level, professional rank, career category, risk perception, and exposure to COVID-19 cases were significantly associated with symptoms of depression (*p* < 0.05); hospital location, health care unit, risk perception, and exposure to COVID-19 cases were significantly associated with symptoms of anxiety (*p* < 0.05); and gender, risk perception, and exposure to COVID-19 cases were significantly associated with symptoms of stress (*p* < 0.05).

**Table 4:** Univariate analysis of symptom of depression, anxiety, and stress.

	**Depression (mild or higher category)**				**Anxiety (mild or higher category)**				**Stress (mild or higher category)**			
	***n***	**%**	**χ^2^**	***p***	***n***	**%**	**χ^2^**	***p***	***n***	**%**	**χ^2^**	***p***
**Hospital location**			20.399	< 0.001			12.971	< 0.001			3.044	0.081
County	542	21.6			645	25.7			393	15.7		
Urban	253	29.2			278	32.1			158	18.2		
**Gender**			2.274	0.132			1.277	0.258			7.901	0.005
Male	257	25.2			292	28.7			194	19.1		
Female	538	22.8			631	26.8			357	15.2		
**Age**			2.039	0.361			0.208	0.901			1.372	0.504
18–30	186	22.4			227	27.3			127	15.3		
31–45	438	24.6			484	27.1			292	16.4		
>45	171	22.6			212	28.0			132	17.4		
**Marital status**			0.779	0.378			0.225	0.636			0.128	0.720
Unmarried/Divorced/widowed	169	24.9			191	28.1			108	15.9		
Married	626	23.3			732	27.2			443	16.5		
**Education level**			19.732	< 0.001			3.870	0.144			2.448	0.294
Junior college or below	221	21.9			272	26.9			177	17.5		
Bachelor's degree	473	22.9			555	26.9			321	15.5		
Master degree or above	101	33.9			96	32.2			53	17.8		
**Health care unit**			12.081	0.060			18.203	0.006			10.439	0.107
Internal Medicine	299	37.6			352	38.1			210	38.1		
Surgery	160	20.1			188	20.4			111	20.1		
Obstetrics and Gynecology	44	5.5			50	5.4			23	4.2		
Pediatrics	31	3.9			29	3.1			19	3.4		
Chinese medicine	35	4.4			39	4.2			22	4.0		
Public health section	0	0			1	0.1			1	0.2		
Other sections (Laboratory, etc.)	226	28.4			264	28.6			165	29.9		
**Professional rank**			14.176	0.001			4.074	0.130			3.939	0.140
Junior/No	311	20.8			389	26.0			225	15.0		
Middle	281	27.2			283	27.4			174	16.8		
Senior	203	24.1			251	29.8			152	18.1		
**Working years**			1.116	0.572			0.105	0.949			1.317	0.518
< 10	356	23.3			419	27.4			238	15.6		
10–20	278	24.6			306	27.1			189	16.7		
>20	161	22.5			198	27.7			124	17.4		
**Average monthly income(CNY)**			0.084	0.772			0.194	0.659			0.080	0.777
≤ 5000	500	23.4			579	27.1			346	16.2		
>5000	295	23.8			344	27.8			205	16.6		
**Career category**			8.966	0.003			3.697	0.054			1.573	0.210
Doctor	542	25.2			612	28.5			364	16.9		
Nurse	253	20.7			311	25.4			187	15.3		
**Risk perception**			223.402	< 0.001			206.479	< 0.001			235.382	< 0.001
≤ 3	514	18.5			618	22.3			328	11.8		
>3	281	47.1			305	51.2			223	37.4		
**Exposure to COVID-19 cases**			7.756	0.005			17.742	< 0.001			5.209	0.022
No	709	23.0			815	26.4			491	15.9		
Yes	86	30.3			108	38.0			60	21.1		

### Factors associated with symptoms of depression, anxiety, and stress

The results of correlation analysis and VIF showed that there was no multicollinearity between the independent variables (Supplementary material).

Multivariate logistic regression results as shown in Table 5 revealed that healthcare workers in urban public hospitals (AOR = 1.41, *P* = 0.001), those with a master's degree or higher (AOR = 1.56, *P* = 0.012), those with a mid-level rank (AOR = 1.43, *P* = 0.003), and those with high-risk perceptions (AOR = 4.12, *P* < 0.001) were more likely to suffer from depression. Nurses (AOR = 0.80, *P* = 0.037) were less likely to develop depression than physicians; healthcare workers in urban public hospitals (AOR = 1.35, *P* = 0.002), those with high-risk perception (AOR = 3.68, *P* < 0.001), and those in contact with COVID-19 patients (AOR = 1.53, *P* = 0.002) were more likely to have anxiety disorders; healthcare workers in urban public hospitals (AOR = 1.26, *P* = 0.048), those with high-risk perceptions (AOR = 4.45, *P* < 0.001) were more likely to suffer from stress, while women (AOR = 0.75, *P* = 0.021) were less likely to suffer from stress than men.

**Table 5:** Factors associated with symptom of depression, anxiety, and stress.

	**Depression (mild or higher category)**			**Anxiety (mild or higher category)**			**Stress (mild or higher category)**		
	**AOR**	**95%CI**	***p***	**AOR**	**95%CI**	***p***	**AOR**	**95%CI**	***p***
**Hospital location**									
County (reference)									
Urban	1.41	1.16–1.71	0.001	1.35	1.12–1.62	0.002	1.26	1.00–1.57	0.048
**Gender**									
Male (reference)									
Female	0.99	0.80–1.23	0.939	1.01	0.82–1.24	0.915	0.75	0.58–0.96	0.021
**Age**									
18–30 (reference)			0.520			0.642			0.627
31–45	0.90	0.68–1.19	0.466	0.90	0.69–1.18	0.453	0.93	0.67–1.29	0.669
>45	0.78	0.52–1.19	0.253	0.83	0.56–1.23	0.358	0.80	0.50–1.28	0.351
**Marital status**									
Unmarried/Divorced/widowed (reference)									
Married	0.81	0.64–1.03	0.088	0.91	0.73–1.14	0.428	0.94	0.71–1.23	0.651
**Education level**									
Junior college or below (reference)			0.007			0.320			0.186
Bachelor's degree	0.97	0.79–1.19	0.741	0.93	0.77–1.13	0.492	0.83	0.66–1.04	0.103
Master degree or above	1.56	1.10–2.20	0.012	1.15	0.82–1.62	0.405	0.99	0.66–1.49	0.962
**Health care unit**									
Internal medicine (reference)			0.824			0.039			0.248
Surgery	0.98	0.77–1.25	0.868	0.97	0.77–1.22	0.813	0.87	0.66–1.14	0.315
Obstetrics and Gynecology	0.81	0.55–1.18	0.266	0.74	0.52–1.05	0.095	0.58	0.36–0.94	0.027
Pediatrics	0.78	0.50–1.20	0.260	0.56	0.36–0.87	0.010	0.71	0.42–1.19	0.192
Chinese medicine	0.88	0.58–1.35	0.572	0.85	0.57–1.27	0.429	0.78	0.47–1.28	0.320
Public health section	0.00	0.00	0.998	0.14	0.02–1.10	0.062	0.29	0.04–2.24	0.233
Other Sections (Laboratory, etc.)	0.89	0.72–1.10	0.294	0.84	0.69–1.02	0.078	0.89	0.70–1.12	0.317
**Professional rank**									
Junior/No (reference)			0.012			0.222			0.405
Middle	1.43	1.13–1.81	0.003	1.08	0.86–1.35	0.503	1.14	0.87–1.49	0.340
Senior	1.33	0.96–1.84	0.082	1.30	0.96–1.76	0.087	1.28	0.89–1.83	0.188
**Working years**									
< 10 (reference)			0.844			0.955			0.914
10–20	1.07	0.84–1.35	0.579	0.97	0.77–1.21	0.777	1.04	0.80–1.37	0.761
>20	1.03	0.72–1.46	0.889	0.96	0.69–1.34	0.806	1.09	0.73–1.63	0.679
**Average monthly income (CNY)**									
≤ 5000 (reference)									
>5000	0.87	0.71–1.07	0.176	0.92	0.75–1.11	0.372	0.91	0.72–1.15	0.418
**Career category**									
Doctor (reference)									
Nurse	0.80	0.64–0.99	0.037	0.86	0.70–1.05	0.134	0.99	0.78–1.27	0.954
**Risk perception**									
≤ 3 (reference)									
>3	4.12	3.40–5.00	0.000	3.68	3.05 −4.44	0.000	4.45	3.62–5.47	0.000
**Exposure to COVID−19 cases**									
No (reference)									
Yes	1.23	0.93–1.64	0.150	1.53	1.17–2.00	0.002	1.21	0.88–1.67	0.247

## Discussion

The purpose of this study was to assess the mental health problem among physicians and nurses in Jilin Province, China, during a period of regular COVID-19 epidemic prevention and control. The COVID-19 epidemic in China was sporadically distributed across several regions, while Jilin Province had no confirmed COVID-19 cases or deaths for seven consecutive months, indicating a relatively stable epidemic situation in the region during the investigation. There is substantial evidence in the previous literature that healthcare workers may have a considerable burden of psychological distress during an outbreak, which has a significant impact on their mental health, outbreak prevention and control efforts, and healthcare decisions (10, 41).

Our study showed that the estimated prevalence rates of depression, anxiety, and stress symptoms were 23.6, 27.4, and 16.3%, respectively, in the population as a whole. A study conducted by Teris Cheung in 2015 on nurses in Hong Kong, China, showed that 35.8% of participants had a prevalence of depression, 37.3% had symptoms of anxiety and 41.1% had symptoms of stress. In their study, the results of depression, anxiety and stress were all higher than those in our study (42). In addition, the level of emotional distress among healthcare workers was lower in our study compared to an early 2020 study (13, 43). The first month of the COVID-19 study conducted by Benedetta Demartini in Italy showed that 41.5% of the population experienced pathological depression, 38.2% experienced anxiety, and 48% experienced stress (43). The results study conducted in Wuhan, China in early 2020 were 50.4% for depression, 44.6% for anxiety, and 71.5% for stress (13). However, the results of the present study differ from those of previous studies following infectious disease epidemics. Lee et al.'s study showed that SARS survivors exhibited worrying levels of psychological stress 1 year after the SARS outbreak, manifesting alarmingly high levels of depression, anxiety, and posttraumatic symptoms, as well as high rates of potential cases of psychiatric disorders (17). Lee suggests that the results may be related to concerns about the complications of SARS and its treatment, economic issues or stigma. We suggest that the results of this study may be related to the stage of development of the COVID-19 epidemic. The gradual control of the epidemic with appropriate government intervention and the reduction of patients could improve the psychological state by reducing the stress of security threats to medical personnel (44). In addition, adequate protective equipment and experience in prevention and control may also contribute to the psychological relief of health care workers.

We found that the prevalence of depression and anxiety was significantly higher in the physician population than in the nurse population in Jilin Province in the context of a seven-month period with no new cases of Covid-19 during normative prevention and control (depression, 25.2% for physicians vs. 20.7% for nurses; anxiety, 28.5% for physicians vs. 25.4% for nurses, *p* < 0.05), which differs from the results of many other previous related studies (21–23). Some studies suggest that the nature of nurses' work, which requires them to be in close contact with patients and to work longer hours, can lead to more severe mental health problems (13, 22, 23). However, some studies show similarities to our results. In other studies, increased mental health symptoms amongst physicians were attributed to burnout caused by the dual stress of the physician population needing to assess and diagnose patients and the stress of COVID-19 infection. Burnout is a state of physical and mental exhaustion that occurs as a result of being in an emotionally demanding work environment for a long period (45). Maslach et al. described burnout as a three-dimensional syndrome consisting of emotional exhaustion, personal depersonalization, and reduced personal accomplishment (46). Numerous previous studies have confirmed the correlation between burnout and depressive symptoms in medical professionals (47). The results of a survey conducted during the Spanish outbreak showed that physicians experienced higher levels of burnout than nurses, possibly related to the added stress on physicians in a crisis needing to make quick, correct decisions amongst unknown factors (48). This is supported by our study's finding that medical professionals with master's degrees or higher and mid-level titles were more likely to experience depressive symptoms. This is likely because related studies have shown that education and job title have an impact on burnout among healthcare workers (49, 50).

The multivariate logistic regression results of this study showed that risk perception was a significant factor that influenced depression, anxiety, and stress in healthcare workers and that healthcare workers with high-risk perception were 3–4 times more likely to suffer from depression, anxiety, and stress than those with low-risk perception. Previous studies have also found a strong correlation between perceived risk and emotional distress in the context of COVID-19, which is consistent with other studies during the pandemic. According to Slovic's psychometric paradigm, there are two main dimensions of risk perception: “fear” and “unknown” (27). The occurrence of a public health emergency is likely to stimulate these two psychological dimensions in people; the perception of risk drives emotional reactions and psychological distress. According to social stress theory, the threat of COVID-19 may trigger significant stress in groups, leading to high levels of risk perception, which may lead to mental health problems (51). In addition, studies have shown that individuals' subjective perceptions of risk may not be consistent with the objective situation. Therefore, it is necessary to focus on the subjective risk perceptions of medical personnel regarding COVID-19 infection during the stabilization of the epidemic, and guide them to maintain a correct and positive subjective perception of the risk of COVID-19 infection, thereby alleviating emotional distress and improving mental health problems of medical personnel.

One interesting observation from our study is that although exposure to COVID-19 patients was associated with depression, anxiety, and stress in the univariate analysis, medical staff exposed to newly diagnosed patients were more likely to suffer from anxiety after controlling for other variable interference, while depression and stress did not demonstrate significant differences, which may be related to the long period without new cases in Jilin Province during the survey period, while the national epidemic continued to emerge. We also found that health professionals with a master's degree or higher were more likely to suffer from depression, which is inconsistent with other results (52) and may be related to the fact that those with higher education among health professionals tend to take on heavier workloads and decision-making tasks. In addition, consistent with other studies, we found that medical staff in urban public hospitals were more likely to suffer from depression and anxiety symptoms, which may be related to the higher workload of urban medical staff compared to county medical staff and the higher number of anti-epidemic tasks supporting other provinces with epidemics. In contrast to previous studies, our study found that men were more likely to suffer from stress symptoms than women, which may be related to the fact that the Chinese physician population is predominantly male.

This study has some limitations. First, because this study used a cross-sectional design, no inferences can be made about the causal relationships of the variables. Second, some healthcare workers supported other cities during the survey, which may have led to some bias in the sampling. Third, due to the large variation in the number of healthcare workers between hospitals, we used the quota sampling method. However, it is non-probability sampling and has limitations in terms of sample representativeness. Fourth, according to Jilin Statistical Yearbook (2021), a total of 212,140 health technical personnel and 3,066,700 hospital admissions in Jilin Province in 2020. The ratio is 0.069. Previous studies have mentioned shortage of health human resources as a cause of increased stress and mental health problems during the COVID-19 pandemic and an exploration of the shortage of human resources for health is lacking in our study. Future studies may need to consider further research on the mental health of healthcare workers in prolonged epidemics through longitudinal studies, probability sampling methods and consideration of human resources for health issues.

## Conclusion

Compared with those reported during the early outbreak of COVID-19 in the early 2020s, mental health problems among healthcare workers were lower in a stable prevention-and-control situation and corroborated converged recent national and international studies. Physicians were more likely to suffer from depression than nurses. Risk perception was highly predictive of depression, anxiety, and stress symptoms among medical personnel.

## Data availability statement

The datasets presented in this article are not readily available because the datasets are currently used for another project, but are available from the corresponding author on reasonable request. Requests to access the datasets should be directed to XY, xhyu@jlu.edu.cn.

## Author contributions

Conceptualization: XY and LN. Methodology: HJ and LN. Formal analysis and investigation: SG, PS, and PC. Writing—original draft preparation: LN. Writing—review and editing: JY and HJ. Funding acquisition and supervision: XY. All authors contributed to the article and approved the submitted version.

## Conflict of interest

The authors declare that the research was conducted in the absence of any commercial or financial relationships that could be construed as a potential conflict of interest.

## Publisher's note

All claims expressed in this article are solely those of the authors and do not necessarily represent those of their affiliated organizations, or those of the publisher, the editors and the reviewers. Any product that may be evaluated in this article, or claim that may be made by its manufacturer, is not guaranteed or endorsed by the publisher.
