# Supplementary material for: Mental health among healthcare workers during the prolonged COVID-19 pandemic: A cross-sectional survey in Jilin Province in China
Source: Front Public Health. 2022 Oct 17;10:1030808. doi: 10.3389/fpubh.2022.1030808 (PMC9618943; doi:10.3389/fpubh.2022.1030808)
Supplement: Supplementary file 1 [file Data_Sheet_1.docx]

Supplementary Material

Supplementary material 1: Results of correlation analysis

**Table S1 Correlation analysis**

|  | **Hospital location** | **Gender** | **Age** | **Marital status** | **Education level** | **Professional rank** | **Working years** | **Average monthly income** | **Career category** | **Risk perception** | **Exposure to COVID-19 cases** |
| --- | --- | --- | --- | --- | --- | --- | --- | --- | --- | --- | --- |
| **Hospital location** | 1 |  |  |  |  |  |  |  |  |  |  |
| **Gender** | 0.054^**^ | 1 |  |  |  |  |  |  |  |  |  |
| **Age** | -0.069^**^ | -0.108^**^ | 1 |  |  |  |  |  |  |  |  |
| **Marital status** | -0.051^**^ | -0.026 | 0.402^**^ | 1 |  |  |  |  |  |  |  |
| **Education**  **level** | 0.248^**^ | -0.076^**^ | -0.101^**^ | 0.040^*^ | 1 |  |  |  |  |  |  |
| **Professional rank** | -0.034^*^ | -0.087^**^ | 0.667^**^ | 0.297^**^ | 0.093^**^ | 1 |  |  |  |  |  |
| **Working years** | -0.095^**^ | -0.002 | 0.724^**^ | 0.309^**^ | -0.130^**^ | 0.637^**^ | 1 |  |  |  |  |
| **Average monthly income** | 0.072^**^ | -0.139^**^ | 0.392^**^ | 0.152^**^ | 0.177^**^ | 0.468^**^ | 0.335^**^ | 1 |  |  |  |
| **Career category** | -0.023 | 0.460^**^ | -0.160^**^ | -0.023 | -0.206^**^ | -0.170^**^ | 0.007 | -0.156^**^ | 1 |  |  |
| **Risk perception** | -0.007 | -0.007 | 0.082^**^ | 0.058^**^ | -0.059^**^ | 0.071^**^ | 0.078^**^ | 0.001 | 0.001 | 1 |  |
| **Exposure to COVID-19 cases** | 0.078^**^ | -0.019 | 0.081^**^ | 0.038^*^ | 0.078^**^ | 0.106^**^ | 0.076^**^ | 0.077^**^ | 0.013 | 0.055^**^ | 1 |
| **p<0.01, *p<0.05 | | | | | | | | | | | |

# Supplementary Material 2：Results of linear regression analyses

**Table S2. Results of linear regression models**

| **Dependent variable** | **Independent Variable** | **Unstandardized Coefficients** | **95% confidence interval** | | **Standardized Coefficient Beta** | **t** | **p** | **Collinearity statistics** | |
| --- | --- | --- | --- | --- | --- | --- | --- | --- | --- |
|  |  | **B** | **Lower Bound** | **Upper Bound** |  |  |  | **Tolerance** | **VIF** |
| Depression | (constant) | 4.249 | 2.777 | 5.721 |  | 5.661 | 0.000 |  |  |
|  | Hospital location | 1.311 | 0.650 | 1.973 | 0.066 | 3.886 | 0.000 | 0.917 | 1.090 |
|  | Gender | -0.505 | -1.190 | 0.180 | -0.027 | -1.446 | 0.148 | 0.776 | 1.289 |
|  | Age | -0.288 | -0.967 | 0.390 | -0.023 | -0.834 | 0.405 | 0.354 | 2.825 |
|  | Marital status | -0.912 | -1.672 | -0.152 | -0.042 | -2.352 | 0.019 | 0.824 | 1.214 |
|  | Education level | 0.340 | -0.188 | 0.867 | 0.023 | 1.263 | 0.207 | 0.803 | 1.245 |
|  | Professional rank | 0.559 | 0.042 | 1.076 | 0.052 | 2.121 | 0.034 | 0.438 | 2.284 |
|  | Working years | 0.055 | -0.503 | 0.613 | 0.005 | 0.193 | 0.847 | 0.406 | 2.465 |
|  | Average monthly income | -0.999 | -1.671 | -0.327 | -0.055 | -2.916 | 0.004 | 0.731 | 1.368 |
|  | Career category | -0.653 | -1.333 | 0.027 | -0.036 | -1.883 | 0.060 | 0.718 | 1.394 |
|  | Risk perception | 7.205 | 6.474 | 7.937 | 0.316 | 19.308 | 0.000 | 0.984 | 1.016 |
|  | Exposure to COVID-19 cases | 0.842 | -0.169 | 1.853 | 0.027 | 1.632 | 0.103 | 0.972 | 1.028 |
| Anxiety | (constant) | 4.599 | 3.071 | 6.127 |  | 5.902 | 0.000 |  |  |
|  | Hospital location | 1.394 | 0.708 | 2.081 | 0.068 | 3.981 | 0.000 | 0.917 | 1.090 |
|  | Gender | -0.409 | -1.120 | 0.302 | -0.021 | -1.129 | 0.259 | 0.776 | 1.289 |
|  | Age | -0.324 | -1.029 | 0.380 | -0.025 | -0.903 | 0.366 | 0.354 | 2.825 |
|  | Marital status | -0.562 | -1.351 | 0.228 | -0.025 | -1.395 | 0.163 | 0.824 | 1.214 |
|  | Education level | -0.064 | -0.612 | 0.483 | -0.004 | -0.230 | 0.818 | 0.803 | 1.245 |
|  | Professional rank | 0.437 | -0.100 | 0.973 | 0.039 | 1.596 | 0.111 | 0.438 | 2.284 |
|  | Working years | 0.158 | -0.422 | 0.737 | 0.014 | 0.533 | 0.594 | 0.406 | 2.465 |
|  | Average monthly income | -0.510 | -1.208 | 0.188 | -0.027 | -1.433 | 0.152 | 0.731 | 1.368 |
|  | Career category | -0.393 | -1.099 | 0.312 | -0.021 | -1.093 | 0.274 | 0.718 | 1.394 |
|  | Risk perception | 7.305 | 6.546 | 8.065 | 0.310 | 18.857 | 0.000 | 0.984 | 1.016 |
|  | Exposure to COVID-19 cases | 1.729 | 0.680 | 2.779 | 0.053 | 3.230 | 0.001 | 0.972 | 1.028 |
| Stress | (constant) | 5.429 | 3.786 | 7.072 |  | 6.480 | 0.000 |  |  |
|  | Hospital location | 1.433 | 0.694 | 2.171 | 0.065 | 3.804 | 0.000 | 0.917 | 1.090 |
|  | Gender | -0.844 | -1.609 | -0.079 | -0.040 | -2.164 | 0.031 | 0.776 | 1.289 |
|  | Age | -0.282 | -1.040 | 0.475 | -0.020 | -0.731 | 0.465 | 0.354 | 2.825 |
|  | Marital status | -0.325 | -1.174 | 0.523 | -0.014 | -0.752 | 0.452 | 0.824 | 1.214 |
|  | Education level | -0.118 | -0.707 | 0.470 | -0.007 | -0.394 | 0.694 | 0.803 | 1.245 |
|  | Professional rank | 0.777 | 0.200 | 1.354 | 0.065 | 2.639 | 0.008 | 0.438 | 2.284 |
|  | Working years | -0.098 | -0.721 | 0.526 | -0.008 | -0.307 | 0.759 | 0.406 | 2.465 |
|  | Average monthly income | -0.569 | -1.319 | 0.181 | -0.028 | -1.488 | 0.137 | 0.731 | 1.368 |
|  | Career category | -0.368 | -1.127 | 0.390 | -0.018 | -0.952 | 0.341 | 0.718 | 1.394 |
|  | Risk perception | 7.762 | 6.945 | 8.578 | 0.306 | 18.633 | 0.000 | 0.984 | 1.016 |
|  | Exposure to COVID-19 cases | 1.093 | -0.035 | 2.222 | 0.031 | 1.899 | 0.058 | 0.972 | 1.028 |
